# Considerations of Australian community pharmacists in the provision and implementation of cognitive pharmacy services: a qualitative study

**DOI:** 10.1186/s12913-021-06838-x

**Published:** 2021-09-03

**Authors:** Faith R. Yong, Su-Yin Hor, Beata V. Bajorek

**Affiliations:** 1 Discipline of Pharmacy, Graduate School of Health, Faculty of Health, University of Technology Sydney, 100 Broadway, Chippendale, Sydney, NSW 2008 Australia; 2 Centre for Health Services Management, Faculty of Health, University of Technology Sydney, Building 10, 15 Broadway, Ultimo, Sydney, NSW 2007 Australia

**Keywords:** Health services, Implementation, Translation, Role, Pharmacist

## Abstract

**Background:**

Australian federally-funded cognitive pharmacy services (CPS) (e.g. medication management and reconciliation services) have not been translated into practice consistently. These health services are purportedly accessible across all Australian community pharmacies, yet are not delivered as often as pharmacists would like. There are international indicators that pharmacists lack the complete behavioural control required to prioritise CPS, despite their desire to deliver them. This requires local investigation.

**Objective:**

To explore Australian pharmacists’ perspectives [1] as CPS providers on the micro level, and [2] on associated meso and macro level CPS implementation issues.

**Methods:**

Registered Australian community pharmacists were recruited via professional organisations and snowball sampling. Data were collected via an online demographic survey and semi-structured interviews until data saturation was reached. Interview transcripts were de-identified then verified by participants. Content analysis was performed to identify provider perspectives on the micro level. Framework analysis using RE-AIM was used to explore meso and macro implementation issues.

**Results:**

Twenty-three participants across Australia gave perspectives on CPS provision. At the micro level, pharmacists did not agree on a single definition of CPS. However, they reported complexity in interactional work and patient considerations, and individual pharmacist factors that affected them when deciding whether to provide CPS. There was an overall deficiency in pharmacy workplace resources reported to be available for implementation and innovation. Use of an implementation evaluation framework suggested CPS implementation is lacking sufficient structural support, whilst reach into target population, service consistency and maintenance for CPS were not specifically considered by pharmacists.

**Conclusions:**

This analysis of pharmacist CPS perspectives suggests slow uptake may be due to a lack of evidence-based, focused, multi-level implementation strategies that take ongoing pharmacist role transition into account. Sustained change may require external change management and implementation support, engagement of frontline clinicians in research, and the development of appropriate pharmacist practice models to support community pharmacists in their CPS roles.

**Trial registration:**

This study was not a clinical intervention trial. It was approved by the University of Technology Sydney Human Research Ethics Committee (UTS HREC 19–3417) on the 26th of April 2019.

## Background

In Australia, pharmacists practising in a pharmacy in a community setting (e.g. shopping centres, retail strips, etc.) are considered ‘community pharmacists’. During their practice, they are required to gather clinical information about their patients through interviewing and counselling processes. These pieces of information, or ‘considerations’, are used to inform their clinical decision-making, enabling patient consultations to be tailored according to the individual’s medication experiences and needs. A previous scoping review found that the work completed by community pharmacists included many more of these ‘considerations’ than previously acknowledged [1]. In this paper, we use the term ‘considerations’ in a similar way, as information that informs pharmacists’ decision-making regarding the provision of newer health services in their workplaces.

The diversification of health services that these Australian community pharmacists provide has slowly and steadily increased in preceding decades. This has arisen from (a) changes in societal demands for accessible health care, (b) a need for differentiation of pharmacy services in the community sector, and (c) new applications within the scope of pharmacist knowledge and associated research. As accessible primary care health professionals, community pharmacists are ideally placed to provide medication and health management services that complement general practitioner and allied health service offerings. This decreases the general public demand for services in crowded emergency departments and overworked medical clinics, and is especially important during health crises such as the COVID-19 pandemic. The resultant health services are known generally as ‘services’ within the Australian community pharmacy sector (as opposed to dispensing and related activities), with several terms and definitions used in the industry (see Table 1) [6]. For consistency, these newer health services will be referred to in this paper as ‘cognitive pharmacy services’ (CPS).

**Table 1:** Pharmacy service definitions

Term	Definition
Community pharmacy services	Every health service provided by a community pharmacy in Australia.
Professional pharmacy services	“An action or set of actions undertaken in or organised by a pharmacy, delivered by a pharmacist or other health practitioner, who applies their specialised health knowledge personally or via an intermediary, with a patient/client, population or other health professional, to optimise the process of care, with the aim to improve health outcomes and the value of healthcare.” [2]
Cognitive pharmacy services (CPS)	Pharmacy services where translation of pharmacist knowledge is the primary characteristic of the task, rather than medication supply [3].
Enhanced pharmacy services	Services “within the current scope of community pharmacy practice and require no additional specific qualifications or credentialing, although additional training specific to that service may be part of its implementation” [4, 5].
Extended pharmacy services	Services which are “an extension of the existing scope of community pharmacy practice and require additional credentialing education and qualifications”, such as vaccination [4, 5].
Community Pharmacy Agreement services	Services funded by the Federal Government through five-year Community Pharmacy Agreements (CPAs), which are negotiated with the Pharmacy Guild of Australia and recently the Pharmaceutical Society of Australia. These services are usually restricted to certain populations by patient eligibility criteria, and funded per service quantity. This funding normally goes directly to a pharmacy organisation, rather than the pharmacist provider [3]. These services are abbreviated according to the CPA number: e.g. 7CPA services refers to Seventh CPA services.

Many of the newer pharmacy services, such as influenza immunisation and home medication reviews, are supported by research studies [2–5, 7–9] which have provided clinical, humanistic, and economic evidence to support their implementation into practice, resulting in their inclusion as remunerated services under five-year federal Community Pharmacy Agreements (CPAs) [10]. These community pharmacy services are funded by the Federal Government under these CPAs as negotiated with the Pharmacy Guild of Australia (‘the Guild’) [11] and, more recently, the Pharmaceutical Society of Australia (PSA) [10, 12]. Specific patient eligibility criteria limit the funding for certain populations, and are paid per service quantity delivered. This typically is paid to a pharmacy, rather than pharmacist providers [13–16]. The securement of this federal funding to sustain the implementation of these services was hailed as a landmark decision for the Australian pharmacy landscape by the pharmacy profession [15, 17], with much applause for those pharmacy academics who had conceived and driven CPS trials [10].

Yet, despite this apparent breakthrough in the delivery of pharmacy-led services, CPS uptake has been relatively slow and/or fragmented; despite projections of high uptake in the community, many CPS implemented and funded in Australia have not met expectations [12, 13, 18, 19]. This is despite pharmacists both approving of and being motivated to provide CPS [20, 21]. International literature suggests that despite preferring to provide CPS [22–24], community pharmacists may not have the freedom to do so [25, 26] and thus prioritise more traditional expectations such as dispensing-related services [6, 27–30]. This evidence suggests that CPS have not been completely implemented in the Australian community pharmacy setting.

Thereby from an implementation perspective, incomplete adoption of CPS across Australian pharmacies could be due to:

1. Poor translation and uptake of research into practice,
2. A lack of targeted implementation and change management processes to address complex community pharmacy settings,
3. Insufficient structural support for pharmacy businesses and pharmacist role change, and
4. Lack of tailored, evidence-based implementation strategies in pharmacy practice and related research.

It could be contended that the transfer of various CPS from trial to practice has been inadequately managed in the Australian setting. Industry groups like the Guild and PSA have argued that pharmacists are already well equipped to provide CPS [31, 32]. This could explain why research trial components like pre-implementation credentialling may not be similarly adopted in actual practice. For example, a federally funded Australian research trial targeting patients with type 2 diabetes, the Diabetes Management and Assessment Service (DMAS), was conducted under the Third Community Pharmacy Agreement (3CPA) [33]. The trial included a two-day training workshop, followed by competency assessments for credentialing for participating pharmacists, and comprised five patient consultations using evidence-based interventions, resulting in increased adherence and a statistically significant blood glucose reduction [33]. However, rather than including this locally validated DMAS as a funded CPS, in the subsequent Fifth Community Pharmacy Agreement (5CPA) the Diabetes Medscheck program [18] was included instead, based on the Canadian Diabetes Medscheck, with less established evidence of benefit [34]. The Guild appears to have adapted these protocols for Australia [13, 15, 35], wherein no specific pharmacist training was required to become a provider. Program evaluations of the Diabetes Medscheck program did not focus on clinical data [18, 36], although pharmacist training was recommended. Subsequent voluntary online education by the PSA did not cover DMAS competencies of motivational interviewing, collaborative goal setting or patient education strategies, nor include requisite credentialing [18, 33, 36, 37]. In the 2017–18 financial year, the annual numbers of Diabetes Medschecks provided were largely similar to the low service delivery observed in the Australian pilot Medscheck study – a poor uptake (59,855 Diabetes Medschecks, or around 6% of the Australian population with Type 2 Diabetes) [18, 38, 39], which could be related to its service quality. This questionable selection of service protocol to be implemented demonstrates how implementation of locally validated research trial protocols may be rejected in translation to actual practice due to industry organisation input, regardless of the research evidence. Similar issues are likely to exist for other CPS provided in the community pharmacy setting, wherein neither structured change management nor evidence-based implementation processes are common.

Pharmacies in Australia implementing CPS are likely to have done so due to a change in professional focus [29–31], and out of necessity. A limited and diminishing pool of federal funding for dispensing services [17] has affected pharmacy viability [40, 41]. As a result, pharmacies often deliver multiple CPS which were hastily adopted. These CPS are seen as complementary and supplementary to prescription dispensing services, and in practice are delivered at varying levels of quantity and quality [18, 40–46]. The perceived urgency caused by a decreased pharmacy profitability means pharmacies may not have expertise nor time to trial, resource and embed each new service sustainably. Although implementation programs with tailored strategies could be utilised in the community pharmacy setting [42], it appears that pharmacists without change management expertise have been expected to do so in the complex community pharmacy setting [1].

In the busy world of community pharmacy, pharmacists and staff may be requested to provide a new service without being *resourced* to do so [40, 43–46]. This lack of resources can lead to worker strain and turnover [1, 41], since each CPS has its own set of administrative tasks for claiming federal funding, protocols, guidelines and a body of assumed knowledge from its initial authors [47–50]. Negative consequences caused by under-resourced CPS implementation and provision could be a major barrier to organisational sustainability and worker motivation to deliver them.

Given that pharmacists are well trained to work using a clinical protocol to deliver health services and have access to federal funding to support these, there is a need to further explore why their CPS delivery has been relatively suboptimal. Government reports indicate consumer needs, which CPS were designed to address, have not been fully met [13, 18, 36], highlighting that pharmacy uptake has been less than expected. As supervising clinicians, pharmacists are expected to manage pharmacy workload changes through task delegation to pharmacy support staff [51], a sense of ‘professionalism’ [52] and organisational changes (such as store layout renovation and workflow optimisation) [53]. However, some studies suggest these strategies could only increase pharmacist perceived task performance (i.e. how well they believed they had completed a task) if the supervisory role (‘monitoring demands’) of pharmacist work are taken into account [46, 54]. This suggests the necessity of examining multiple roles that pharmacists enact within a community pharmacy “store” environment [24, 55–57]. Therefore, the structure of the pharmacist role itself has been implicated as a potentially major factor in pharmacist behaviour, whose low rate of CPS delivery is inconsistent with their reported desire to provide CPS [1]. This is important since Australian *pharmacists* are largely responsible for CPS delivery, which by definition requires the use of pharmacist knowledge (rather than pharmacy support staff) [58, 59]. It is therefore important to understand how the professional identities of pharmacists, who may direct, manage and provide the CPS in Australian community pharmacies, can affect CPS implementation. Professional identity could be an important predictor of service quality and fidelity [1, 55, 60], especially in CPS relying on professional knowledge and skills.

Lastly, one review reported that researchers applying CPS implementation trials in community pharmacy have not had strong rationale for their choice of implementation strategies, with low ratings for methodological quality and evidence of outcome quality [61]. Wider reading of implementation science literature outside pharmacy practice suggests the concepts of ‘clinical intervention’ and ‘implementation intervention’ may have been confused in the process of applying implementation science to CPS [62, 63]. Additionally, few true controlled implementation trials in pharmacy practice are being undertaken to analyse the effect of implementation strategies used; only one study using change management was found to address this in Australian community pharmacies [64]. Many implementation science studies in pharmacy practice should more accurately be termed intervention trials, since they aim to prove clinical/economic benefits of new health interventions, rather than the implementation process [65, 66]. This, coupled with the high likelihood that few Australian pharmacies use *any* implementation strategies, project a poor prognosis for CPS implementation in Australian community pharmacy practice.

Thus, on micro, meso and macro levels, there have been fundamental issues with both providing and implementing CPS in Australia. (The ‘micro’ level refers to individual community pharmacists; the ‘meso’ level refers to the community pharmacy business (or network of businesses); and the ‘macro’ level is the community pharmacy sector as part of the broader health care system (including regulatory bodies and professional organisations) [67].) It could be that a top-down implementation approach employed by pharmacy organisations to implement CPS in pharmacies, which are effectively small businesses, has been neither sufficient nor responsive to the patient populations they aim to reach.

Therefore, this article aims, to explore current CPS translation and provision in the Australian community pharmacy setting, and understand related implementation issues, from the pharmacist provider’s perspective. Since previous studies have reported barriers and facilitators in CPS implementation [21, 62, 63, 68, 69], this research was designed to elicit the personal views of community pharmacists as the supervising providers of CPS.

## Objectives

1. To explore the micro level considerations of Australian community pharmacists when deciding to *provide* CPS, including their ability to do so, and
2. To understand meso and macro level issues relating to *implementing* CPS, according to their pharmacist providers.

## Methods

This article used the Consolidated criteria for reporting qualitative research (COREQ) guidelines [70], and Amin et al.’s guidelines [71].

A qualitative semi-structured interview design was used. Questions explored pharmacists’ daily CPS practices and considerations in the delivery of these newer health services, including their personal working preferences and career satisfaction. A pre-interview online survey to capture participant demographics was modified from an UK workforce survey [72], which included age, gender, postcode, multiple jobs worked, working hours and job titles.

In order to explore CPS implementation and provision issues on micro, meso and macro levels, there were two stages of analysis. Firstly, content analysis was employed to explore the micro perspective of the community pharmacists who provide CPS, and may have been responsible for implementation of CPS into pharmacy workflows. The second phase of framework analysis extracted reported issues on the meso and macro levels, from pharmacist perspectives.

### Participant recruitment, inclusion/exclusion criteria and sample size

Initially, recruitment was through purposive and snowball sampling. The pharmacists working in awarded Guild Pharmacy of the Year winners and runners-up from 2016 to 2019 were invited by email to participate. These Pharmacy of the Year awards typically recognise pharmacy excellence in innovation and CPS provision in Australia through self-nomination, or Guild Branch/Quality Care Pharmacy Program (QCPP) assessor nomination [73]. Given that not all Australian pharmacies provide CPS, inviting pharmacists working at these pharmacies was intentional, since they would be motivated and engaged in CPS provision.

Noting that Australian pharmacists can be difficult to reach due to their long working hours and not being typically engaged with research, the study was also promoted through open invitation recruitment emails/posts to consenting professional associations (Pharmaceutical Society of Australia, the Australian Association of Consultant Pharmacists, Small Pharmacies Group, Professional Pharmacists Australia, Rural Pharmacy Network) and posting on consenting closed social media Facebook groups which Australian pharmacists actively participate in. The study was periodically promoted via these channels until September 2019. The Pharmacy Board of Australia and the Pharmacy Guild of Australia declined to be involved in the recruitment process.

Pharmacists with full registration in Australia for at least 6 months who had current or recent work in the community sector were recruited. Pharmacists who did not have full registration for at least 6 months (e.g. students, interns, non-practising registration), did not have experience working in the community setting within 2015–2019 (the last 5 years, or the current CPA at that time).

A target sample size of at least 15 participants was anticipated. However, recruitment continued until data in the content analysis reached saturation. No sample stratification was deemed necessary due to population heterogeneity.

### Data collection

After receiving participant consent forms via email, participant characteristics were collected using a survey hosted on the University Technology of Sydney (UTS) REDCap platform [74, 75].

This survey and the semi-structured interview guide are available in Appendices 2 and 3.

The interview guide and modified survey was piloted with two academic pharmacists who had extensive experience in the community setting. Adjustments to both were made according to their feedback.

The four sections in the final interview guide are as follows:

1. Icebreaker questions querying what pharmacy services were provided at their main pharmacy workplace;
2. Listing factors they considered during service provision, with prompts if necessary (e.g. workplace factors, staffing, patients/doctors, specific services, etc.);
3. Expression of positive, negative or neutral opinions towards providing services and their personal reasons for these; and
4. What work tasks pharmacists personally preferred, and their opinion of their pharmacist career.

The last two sections on the exploration of pharmacist perspectives on service provision, working preferences and career satisfaction were included, since this could affect prior opinions expressed about CPS.

During the interviews, participants were not provided with a definition of professional pharmacy services, and thereby spoke from their own understanding. When requested for a definition, the researcher specified ‘patient-facing services’.

One researcher, FY, conducted these individual semi-structured interviews using online conferencing software Skype or Zoom, which were audio recorded. Video cameras were turned off before interview recording began.

FY is a doctoral candidate who is a community pharmacist with a Bachelor of Pharmacy (Hons.), trained in an intensive week-long qualitative research course. Prior to interviews, FY made contact with participants to answer their questions about the study, and ensure completion of consent forms and the online survey. Prolonged engagement with the consenting social media communities of fellow pharmacists allowed the researcher to discuss the study objectives at length with individual participants, and establish rapport beforehand. When conducting interviews, her status as a community pharmacist and desire to document front-line clinician work was explained to participants. Participants were asked to explain pharmacist work factors as if to a non-clinician for the purposes of the study. Although the interviewing researcher held previous views from practising as a community pharmacist, the same interview guide was used for all participants. This gave participants the opportunity to voice their perspectives for all the aspects of the study being addressed. To avoid assumptions, participant statements were reflected back to them by the interviewer to confirm their thoughts, or participants were asked to elaborate on their statements. Similarly, field notes taken by FY during the interview were used to clarify participant meaning within interviews, and return to previous conversation points of interest. Interview duration was kept within 45–60 min unless participants agreed to extend interviews.

A machine-learning program, NVivo Transcription, was used to transcribe 15 of the transcripts, which were independently and additionally verified by FY. The remainder of interviews were manually transcribed and de-identified by the same researcher. Participants were emailed their respective anonymised transcripts in Microsoft Word document form, and requested to verify the transcripts for clarity and meaning. They were also asked to confirm the transcripts were sufficiently de-identified.

### Data analysis

Inductive content analysis was performed via coding with NVivo software by FY, in a constructivist, iterative process [76]. This phase of analysis was designed to explore pharmacist views of CPS provision on the micro level, and thus report participant insights and meanings [77] into this type of care practice. All transcripts were read to familiarise the researcher with the content. Key thoughts and ideas were assigned a descriptive summary phrase (i.e. code). As transcripts were read in order of case number, codes were added iteratively and constantly compared to transcript data in a cyclical fashion. Wherever necessary, initial codes were adapted during the coding process, and particularly after all transcripts had been coded: as more details emerged about the topic of the code, they were split into multiple codes; at times codes were renamed; and sometimes codes were combined with others. This was done to clarify and refine findings [77]. When content analysis reached theoretical saturation (i.e. no new codes were apparent) [78], recruitment was halted. The resultant codes were then categorised more broadly for reporting, with input from two pharmacy practice academics.

To explore the translation and implementation of CPS on meso and macro levels from the perspective of the individual pharmacist provider, an implementation framework was used to guide a secondary analysis for all transcripts. Framework method analysis was chosen for its ability to structure the data summarisation/reduction to answer a research question [79], that being, ‘What does this data say about CPS implementation by these pharmacists in Australia?’ Whilst various frameworks for prospective and ongoing implementation evaluations were noted, the RE-AIM framework was chosen for framework analysis as it is designed to assess the translation of health policy and research into practice. It involves five domains: Reach to target population, Efficacy or effectiveness in target population, Adoption, Implementation and Maintenance [80, 81]. Although a qualitative arm of the RE-AIM framework (i.e. RE-AIM PRISM) evaluates the implementation of *one* service, it was not utilised since this study was not analysing *one* service alone; furthermore, the PRISM arm requires the input of target populations, which was beyond the scope of this paper [82]. Since the study aimed to elicit pharmacist perspectives alone on the overall implementation and sustainability of several CPS, the overall constructs of the RE-AIM framework (not the RE-AIM PRISM) were deemed more appropriate.

After selecting the evaluating implementation framework, the transcripts underwent framework method analysis in NVivo, using the RE-AIM framework constructs as coding categories [79]. The data within each of these codes were then arranged thematically, and the results summarised for reporting.

## Results

Twenty-three participants were interviewed via an online platform between May and September 2019, with nearly even proportions of female and male pharmacists.

Non-participants may have been present at interviews since participants participated from places of their own discretion, however, video functionalities were disabled during interviews and presence of third parties could not be verified.

One participant who completed the consent form and survey was not interviewed, due to their lack of availability. Two participants spoke informally about their working conditions after recording was halted. FY obtained their written consent to add the field notes recording this information to the end of their respective transcripts. One participant requested specific information given in the interview to be redacted, but later approved its use.

All interviewed participants were given the opportunity to review, verify and modify their anonymised transcripts, and all approved their transcripts for use in the study. One participant edited their transcript for clarity. Three participants admitted they did not read their full transcript since they had no time or felt embarrassed reading their spoken words in written format. When asked by FY, two participants later gave further written clarification about specific statements after transcript verification. Repeat interviews were not considered necessary.

Under half of the participants had been practising for 10 years or less, and the distribution of geographic representation was similar to Pharmacy Board of Australia statistics [83]. Independent pharmacies and franchise/banner groups were represented in approximately even numbers, and there was one not-for-profit friendly society pharmacy represented. Whilst over half of pharmacies represented (52.2%) were in metropolitan areas, the next largest category (21.7%) were Modified Monash Model (MMM) category 5 [84], which represents small rural towns. (The Modified Monash Model defines Australian locations according to population size and geographic remoteness, and is used in national health workforce distributions.) [84] All socio-economic disadvantage indices were represented by the participants, with the most disadvantaged category being most highly represented (21.7%). (The Index of Relative Socio-economic Disadvantage (IRSD) for Socio-Economic Indexes for Areas (SEIFA) is used in Australia to examine relationships between socio-economic disadvantage and health/educational outcomes.) [85] See Table 2 for participant characteristics, and Appendix 1 for a summary of overall demographic statistics.

**Table 2:** Participant characteristics

Participant	Pharmacist position	Age range	Community pharmacy type	IRSD/MMM/PhAria for main community sector job postcode
**1**	Owner/Hospital staff/Consultant	45–49	Franchise/banner	4/5/4
**2**	Owner	40–44	Independent	1/5/4
**3**	Owner/Consultant/Hospital consultant	35–39	Franchise/banner, independent	3/3/1
**4**	Owner/Academic	40–44	Franchise/banner	4/6/5
**5**	Staff	30–34	Franchise/banner	No IRSD data (bounded by 3–4)/1/1
**6**	Owner/Owner/NGO convener	35–39	Independent, independent	1/1/1
**7**	Owner	60–64	Independent	1/7/6
**8**	Casual staff	35–39	Franchise/banner, franchise/banner, independent	2/1/1
**9**	Staff	25–29	Franchise/banner	5/1/1
**10**	Locum	60–64	Franchise/banner	5/1/1
**11**	Staff	25–29	Franchise/banner	3/1/1
**12**	Locum	35–39	Independent	No IRSD data (next to area with 5)/1/1
**13**	Locum	30–34	Independent	5/1/1
**14**	Owner	30–34	Franchise/banner	1/5/3
**15**	Hospital Staff (former locum)	U-25	N/A	Not given
**16**	Owner	55–59	Independent	2/5/3
**17**	Manager/Translator	30–34	Independent	3/4/3
**18**	Staff	45–49	Franchise/banner	No IRSD data/1/1
**19**	Manager	25–29	Friendly society	2/5/3
**20**	Owner	35–39	Franchise/banner	3/1/1
**22**	Staff/Hospital staff/Casual staff	U-25	Franchise/banner, Independent, franchise/banner	4/1/1
**23**	Professional association/staff	30–34	Independent	?/1/1
**24**	Consultant/Manager	30–34	Franchise/banner	1/1/1

*IRSD* Index of Relative Socioeconomic Index, where 1: Most Disadvantaged, and 5: Least disadvantaged [5]

*PhAria* Pharmacy Access/Remoteness Index of Australia [7]

*MMM* Modified Monash Model, where 1: Metropolitan areas, 2: Regional centres, 3: Large rural towns, 4: Medium rural towns, 5: Small rural towns, 7: Very remote communities [8]

The two phases of analysis produced:

(I). Micro level pharmacist perspectives on CPS provision, and.
(II). Pharmacist perspectives on meso and macro level CPS implementation into the community pharmacy setting.

Overall, pharmacists were concerned with the complexities of providing a broad range of pharmacy services (including dispensary services and CPS) to their clientele at sufficient quality within regulatory, ethical and clinical frameworks, whilst also fulfilling their organisational obligation to operate pharmacies in an economically viable manner. They reported their approaches to CPS implementation as pragmatic, ad hoc and resource-dependent in nature.

### Micro level: pharmacist perspectives on CPS provision

Participating pharmacists commented on the difficulty of listing the many factors in service delivery, and therefore tended to prioritise and categorise them. While their opinions differed on which factors were the most important, they spoke at length about being mindful of the differences between individual patients, the influence of relationships with different role partners, and their ability to work freely within the limitations of their workplace, pharmacist role system and resources available.

#### Services

First, pharmacists had trouble recalling all the services provided in their pharmacy. The reason for this became apparent as they began listing services: there were many services per pharmacy being offered, although it was not obvious from their descriptions which services were more commonly provided, or whether some were provided at all.

Second, there was confusion about the definition of services. Pharmacists generally sought clarification upon the exact definition of clinical pharmacist services, as if wanting to answer ‘correctly’. Some argued that all services provided in a pharmacy were clinical, and others asserted that extended services had always been provided on an informal ad hoc basis.

Third, there was disagreement about the nature of services. Some older pharmacists did not identify newer services such as Medschecks as being particularly different in nature to ‘core’ services: instead, they described these as being a continuation of patient care and activities that they had ‘always done’. Mostly, older pharmacists did not acknowledge the fundamental differences in extended or enhanced services (e.g. Medschecks, immunisation and screening services), many of which are designed to be completed in private counselling rooms away from a busy dispensary environment. Younger pharmacists often commented on how difficult it was to be expected to provide both newer CPS and dispensary-based services, especially as there were insufficient workplace resources to effectively do so.

Fourth, there was a decreased priority for CPS compared to ‘core’ services. Pharmacists described ‘core’ pharmacy services as those associated with dispensing prescriptions, and were often identified as being more ‘important’ than CPS. This was regardless of CPS profitability (which was nonetheless oft described as insufficient).

Fifth, pharmacists placed great importance on the quality of service provided. Pharmacists who had routinised professional pharmacy services in their workflow referred to service quality as an important factor in determining patient demand and pharmacist competence. A few participants advocated for adding value to services using the patient perspective, rather than viewing them as brief ‘add-ons’ to dispensing and medication counselling. ‘Quality’, as they described it, included provision of physiological feedback on patient progress using diagnostic machines for pharmacist-delivered services, standardised guidelines with sufficient numbers of trained pharmacists to deliver a consistent service offering, and different funding sources other than government rebates: i.e. patient-paid services, workplace and health insurance funding and rebates. Several pharmacists detailed the various strategies they had trialled in their pharmacies to implement more innovative services. As they spoke, it became obvious that these trials represented years of investment experimenting with various modifications to resources employed including store staffing, the type of pharmacists employed, store layouts and equipment.

#### Patient considerations

The second thematic category related to the patient-based considerations pharmacists in the services offered/provided.

First, pharmacists spoke of being highly conscious of patient differences. They reported being cognisant of, and sensitive to, patient differences and their capability to understand or receive knowledge, particularly as service delivery required negotiating new activities with the patient.

Second, the importance of ‘knowing the patient’ was stressed by several pharmacists. The depth of the pharmacist ‘knowing the patient’ enabled pharmacists to adapt their language, navigate miscommunications and customise services with greater specificity. Many talked about tailoring their service provision by taking into account patient backgrounds, accessibility to health services, language usage, adherence and health literacy.

Third, pharmacists leveraged their relationship with individual patients to address poor or absent doctor-patient dyads.

Particularly in remote areas with the highest Index of Relative Socio-economic Disadvantage (IRSD) score (i.e. most disadvantaged), pharmacists reported helping to mediate and ‘fill in’ for the doctor where the doctor-patient relationship was lacking (e.g. limited access to the doctor, interactions with doctors whom the patient did not know well, had very limited time with, or had difficulties communicating with). Participants gave examples such as: teaching illiterate patients what to do with diagnostic test orders; explanation of disease treatment for patients who could not understand the foreign accents of overseas-trained doctors; case conferencing with general practitioners and specialists to coordinate complex medication regimens for patients living remotely; making tailored collaborative medication decisions with patients, general practitioners and specialists to improve adherence; triaging minor ailments and referring patients for medical interventions when necessary; providing logistic solutions for interstate ‘grey nomad’ travellers requiring medication not readily available or necessitating cold chain storage; acting as stand-in ‘case workers’ for patients on opioid replacement therapy; hiring Aboriginal Health workers to service local Indigenous communities; and a case of identifying possible asthma in a remote-dwelling patient with multiple related hospitalisations. Many of these activities were performed ad-hoc by pharmacists and were reportedly because doctors had either insufficient time with the patient, or did not have a long-term relationship with the patient.

This type of work also appeared to carry into metropolitan pharmacist practice. Pharmacists perceived that patients could be experiencing many different situations such as: having very little time during medical consultations; not knowing if patients could ask questions of busy doctors; or not having a regular general practitioner. However, sometimes they simply knew their pharmacist better. In all of these situations, pharmacist intervention was seen as necessary and significant.

In total, the interactions pharmacists described were highly individualised, and had become possible due to the high level of patient trust and understanding built over years of pharmacist service. The pharmacists saw themselves as ‘middle-men’ between patients and doctors or other healthcare professionals, and generally accepted this role as it meant they could ‘help more people’.

#### The pharmacist themselves

First, positive outcomes motivated pharmacists towards further service innovation. Participants spoke at length about these: increased satisfaction derived from the increased patient satisfaction, contact and health outcomes associated with delivering services. These outcomes led pharmacists to desire extension of these services, allowing them to help more patients.

Second, pharmacists saw their role as important, although other parties did not always recognise what pharmacists had done as significant. Pharmacists perceived their role in the health care system was to be a team player and to ‘fill in’ gaps in patient care where necessary. Where good relationships with doctors were reported, pharmacists spoke of their collaborative efforts with them to take care of patients. When patients and doctors misunderstood or rejected their attempts to protect patient health, pharmacists reported feeling underappreciated and unrecognised for their efforts. This carried into professional pharmacy services, too, where most pharmacists reported dual reasons for providing these services: desiring greater productivity in their preventative health interventions in order to help their patients more, as well as contributing to pharmacy viability. However, misunderstandings of other health professionals and misconceptions of patients in regards to pharmacist qualifications and tasks were cited as a major interactional barrier, impeding provision and implementation of CPS. In other words, patients and doctors were generally less comfortable with pharmacists enacting the clinician role involved in enhanced and extended CPS, compared to medication dispensing and supply roles.

Third, the pharmacist was spoken of as central to the running of the pharmacy. Given the large amount of tasks to be done, many of which could only be completed by registered pharmacists, workforce structuring was presented by pharmacists as a major issue, with a background perception of insufficient organisational funds to support additional pharmacist wages. A minority of participants asserted that a large increase in productivity made wage expenditure worthwhile. One participant spoke of the necessity to use a dispensary technician to direct pharmacy workflow so that the pharmacist would be ‘freed up’ to provide immunisation, Medschecks and other screening services, between checking dispensed items for patient pickup.

#### Pharmacy workplace

The dispensary was spoken of as the pharmacist’s ‘base’, which was associated with multi-tasking, interruptions and distractions. However, this norm seemed to be disrupted by having to deliver CPS like Medschecks, since this required them to leave the dispensary. This meant the multi-tasking and usual work normally completed within dispensary confines could not then be undertaken alongside the CPS, causing a build-up of multiple work tasks and processes which the pharmacist had to return to after completing the CPS.

Second, staffing and store layout were key to service provision. Participants reported that staffing (including the number of pharmacists that could be rostered on) and pharmacy layout (particularly the number of consultation rooms) were limiting factors for expansion of professional pharmacy services. Participants stated bottlenecks in workflow processes were largely due to limited pharmacist availability, who were purportedly needed ‘everywhere’ to do ‘everything’ as the generalists qualified to do all tasks in the pharmacy. The number of counselling rooms, too, were major limiting factors for pharmacies that had succeeded in employing multiple pharmacists on a daily basis. Without these private areas needed for vaccinations and most extended or enhanced services, services could not be provided with guaranteed patient privacy. An apparent lack of privacy afforded was reported to cause patient apprehension and a lack of trust, which could impact on future service demand.

Third, government remuneration had an effect on service provision. Some participants acknowledged the importance of government remuneration in rewarding clinical interventions, which was said to encourage pharmacists in increasingly patient-oriented work approaches. However, remote and regionally located participants reported this remuneration was insufficient, citing high wage overheads needed to attract pharmacists to rural locations and an ‘overwhelming’ workload caused by disparate healthcare service coverage. They reported difficulties in enticing pharmacists from metropolitan areas and a pharmacist workforce shortage. Altogether, this was said to limit the ability of rurally located pharmacies to consistently and formally offer advanced or extended services, since they were already purportedly providing extensive ad-hoc individualised patient services and consultations.

Please see Table 3 for the related participant quotes.

**Table 3:** Quotes for participant themes

Category	Themes	Quotes
Services	Pharmacists had trouble recalling all the services provided in their pharmacy.	*“You know there’s probably the odd service here and now that I’ve forgotten you know. I don’t know.” (Pharmacist owner)* *“Then in terms of other services and trying - just trying to have a run down my brain of what I did last week, because I do so many different things that are like, you can’t pinpoint it because it’s like going from one to another.” (Casual staff pharmacist)*
	Confusion about the definition of services.	*“What I personally provide? Can you be specific on what you call services?” (Pharmacist locum)* *“I don’t know whether or not that’s something that you would consider to be a service in the normal sense of the word.” (Pharmacist owner)* *“I don’t know what you would call that in terms of the realms of services... I don’t know if it’s a service. I mean I see it as a service, because it’s part of what we have.” (Casual staff pharmacist)*
	Disagreement about the nature of services.	*“Medschecks has not been something that I actually actively sit down and get them to sign a paper, but as I said, because I’m old school, you do it, just you’re not - now, I have to think about being paid for them and sometimes I don’t. I don’t. I forget to record them.” (Locum pharmacist)* “*I think a lot of pharmacists - I mean, I could be biased, because I’m a newer one, who’s recently come out of university, but there’s obviously a lot more focus for us to be a lot more hands on and patient interaction and you can see that in the change of pharmacy models with a lot more now coming out from behind the counter.” (Staff pharmacist)*
	Decreased priority for CPS compared to ‘core’ services.	*“So obviously, just like, dispensing and supplying medication is a priority, because that’s what people mainly come in - come in for.”(Staff pharmacist)* *“Because your core business in the current model is still the scripts. […] you can’t say I’m not dispensing today, because it’s part of your Medicare requirement that you’re open and dispensing scripts. […] And I do prioritise the smooth running of the pharmacy over the services – over, over the services that I can say no to, obviously. You know, you have to counsel on new medications. You can’t not.” (Staff pharmacist)*
	Pharmacists placed importance on the quality of service provided.	*“It’s more so, you know, you trial the service, but you go 100% into the service, only to find that because you’re running it properly, the demand is so high. Whereas if you weren’t running it properly, it just, you know, wouldn’t be?” (Staff pharmacist)*
Patient considerations	Highly conscious of patient differences.	*“I find with some people, some people are really happy to hear that [we are offering these services]. Some people actually get a bit nervous when you propose that [they participate in a service], they’re just like “Oh, no, no no, I don’t want to put you to trouble, “or, “I don’t want to.” I think the idea of being in a separate area and we’re sitting down, and like, I think they find it a little bit confrontational, some of them?” (Pharmacist owner)*
	The importance of ‘knowing the patient’.	*“[After implementing extended services] I feel that we, sort of, know our patients even more - a little bit better. So, I like that. I think that’s good… I think that’s, I would say, a definite positive thing for me. ... And I think it’s nice to be able to participate in their health a little bit more. So I’d say it’s much more rewarding.” (Metropolitan pharmacist owner)*
	Pharmacists leveraged their relationship with individual patients to attempt to cover poor or absent doctor-patient dyads: - *In rural/remote areas* - *In metropolitan areas*	*“…yeah there’s three doctors there part time, over the week, but they’re quite busy. So a lot of people - well, the patients come to us, a lot of the time first, if there is something, well, semi-acute, to ask if they really need to go and see them [the doctors].” (Pharmacist owner in small rural town)* *“And it’s not an infrequent for a customer to say to me,,, “Oh thank god, can you explain this to me? Because I couldn’t understand a word of what the doctor’s just said to me. I could not understand the doctor. I was there for 10 min. He said things to me, and I don’t understand. Here’s a script. You explain that to me.”(Pharmacist manager in remote community)* *“…my view of Pharmacy: that we’re the nexus, the connection between the patient and the doctor. Because the doctors don’t have a lot of time, you know, and people always appreciate explanation of their medication, because we have time.” (Pharmacist owner in small remote town)* *“So sometimes doctors miss a lot of details, sometimes doctor’s instructions is not fair. And I have to call, every – like, I have to call a lot of times.” (Staff pharmacist in metropolitan area)* *“I explained to her that the Mersyndol would make her overcoming this addiction harder. And she looked at me and she said, “No one has ever told me that. Not my doctor, no one.”” (Locum pharmacist in metropolitan area)*
Pharmacist	Positive outcomes motivated pharmacists towards further service innovation.	*“…customers have said to me – and they’re saying it constantly to me now, to the point that it sticks in my mind, that, “It’s the most****useful****thing that anyone’s ever done for me in a pharmacy!” (Staff pharmacist)* *“In that way I find it really positive to do service provision and the teaching you get in connection with it, because you get better at explaining things, that you look into more things, makes you a better pharmacist.”(Pharmacist owner)* *“I would like to see remuneration, because I do think that we could do more. And yeah, it’s to enable another pharmacist. … It would mean that we could properly dedicate a time, you know, for example. So we could schedule people in to do, well, the services, that - you know, Medschecks and any queries that come off the streets, so to speak.” (Pharmacist owner)*
	Pharmacists saw their role as important, although others did not always.	*“the number of times a patient, when I explained something to the patient in counselling, and they say, “Why didn’t the doctor tell me that?” You know, if I had a dollar for every time I’ve been told that! And I think that, that’s an important role, because a lot of people go away from the doctors, not knowing a lot that they should know. And that’s the role we play.” (Pharmacist locum)* *“So the local GP was very stressed because she was trying to get him to go to these appointments, because she needed more information. But he couldn’t go. So she and I were trying to manage him, just to keep him safe, in terms of bleeding, and he’s got diabetes and heart failure and he was very complex. …so we were kind of a tag team. Which is what happens with the other doctors as well. They would - they will pass on when a patient leaves, they may ring me and say, ‘Look I spoke about this. Can you - can you please reinforce?’ or, ‘Just for your information, we spoke about this.’” (Pharmacist owner)* *“I think the other healthcare professionals need to understand the role that we’re taking. And I think they need to understand that we are not actually trying to overstep them. We’re trying to offload the pressure that they’re experiencing… and the convenience [for the patient] of [providing extended services] in this day and age.” (Pharmacist manager)*
	The centrality of the pharmacist to the running of the pharmacy.	*“So pretty much everything in the pharmacy stops, unless the pharmacist’s there.” (Staff pharmacist)* *“[…]one pharmacist is worth ten pharmacy assistants…”(Pharmacist owner)*
Pharmacy workplace	The dispensary as the pharmacist’s base.	*“I guess for that, particularly for Medschecks is that, you - if a pharmacist is devoting their time for, whatever it is, say 30 min, three quarters of an hour, to sit down with someone, you know, in a proper counselling room, then they can’t be doing anything else in the pharmacy. And so if someone brings in a prescription during that time or comes in for a OTC medication, that person can’t be served because you can’t be interrupted while you’re doing your Medscheck.” (Pharmacist owner)*
	Staffing and store layout.	*“I think that is, even when we had the right number of staff to do it, we still failed. And it wasn’t until we completely changed the layout of the store, you know, put the automation in that that we began to gain some traction with health services.” (Pharmacist owner)*
	The effect of government remuneration.	*“But when I look at it from a business perspective, if you have to keep the business running, you have to be very careful with where you spend your time, ok? And if you don’t get enough rewarding* …. *you not gonna be able to sustain your business. And that’s when I realise, oh, I see why these incentives are so essential. Because these incentives are the reason why I think, ‘Oh, it’s worth it. It’s not only I am helping patients*; *I am also helping the business.’ So I think incentives is important.” (Staff pharmacist)*

### Pharmacist perspectives on meso and macro levels of CPS implementation into the community pharmacy setting

Regardless of their role in the pharmacy organisation, participants showed themselves to be concerned with CPS implementation. Their strategies to address these were ad hoc and pragmatic rather than evidence-based, demonstrating insufficient support from either professional organisations or federal programs. The more innovative participants had trialled their own implementation strategies, and created symbiotic relationships with chain pharmacy offices and private insurance companies, out of a sense of necessity. However, there was a lack of detail in how populations were to be targeted through CPS, how they measured CPS consistency and any systematic monitoring of CPS maintenance effects in individual patients and their pharmacies. Instead, they focused on their own professional practice, the resources available and how the pharmacy owner administered their business. Whilst some participants reported a more systematic approach towards implementing CPS in general, other modifications were made to the CPS provision itself by making the language more acceptable and comprehensible to clients. Main outcomes pharmacists reported were ad hoc case reports of patient satisfaction or improved health outcomes, and financial return.

See Table 4 for the relevant quotes for each RE-AIM framework construct listed below.

**Table 4:** RE-AIM framework evaluation quotes

Framework construct	Level of impact	Quotes
**Reach to target population**	Individual	*“We don’t have a hospital as such, but we’ve got a local medical service with an attached [nursing home], and, yeah there’s three doctors there part time, over the week, but they’re quite busy. So a lot of people - well, the patients come to us, a lot of the time first, if there is something, well, semi-acute, to ask if they really need to go and see them [the doctors].” (Participant 2, owner)*
**Efficacy or effectiveness in target population**	Individual	*“What makes service provision worth it? Making real improvements to people’s lives. That’s probably the easiest way to say it. I mean you can just say, their health, but when people say health, I think people tend to think of it very clinically. Yeah, but it’s not just about all - their cholesterol levels have come down, their sugar has stabilized. It’s not just about the pathology results. It’s also about the quality of the lives that they have afterwards, improving. You know, like the person who can breathe better because they’ve finally figured out how to use a frickin, like, asthma spray properly.” (Participant 6, owner)* *“So it’s not like we bought the [machine], which is a $1000 machine, thinking, ‘Oh, we’ve got all this market for HbA1c and cholesterol testing.’ We didn’t think that at all! We thought that having this machine would augment our core service, which is the weight loss, and, and it’s ended up – because this was at my previous pharmacy – and it ended up, it ended up being really the thing that people valued, and the thing that really brought them into the pharmacy for the weight loss service. Because they weren’t worried about their weight, they were worried about their health. And so the fact that we could do that for them on the spot, was amazing for them! And so they were saying, “It’s the most useful thing I’ve ever had done for me in the pharmacy.”” (Participant 18, staff)*
**Adoption**	Organisational (staff)	*“…if I had all the resource[s] that I can have, then, what I would do is, I would love to have a pharmacist, or two! Who – who’s just dedicated to, to providing service. To providing clinical services, and things like that. I would love to have it, an outreach pharmacist who can do all the reviews for us, um, and, and - yeah. And if, if pharmacists, if pharmacists can work offsite, or do other things as well, that would be ideal as well. But it’s because of the, of the funding structures. It’s not really adequate. Yeah. So. So you don’t know – you don’t really have the resource to transform that, that’s basically [it].” (Participant 20, owner)*
	Organisational (institutions: i.e. community pharmacies)	*“Diabetes [services], I tend to [provide] as much as possible, if the pharmacy allows me to be customer oriented pharmacy, I do like that. But not every pharmacy, I can do that. Some, some just don’t have any support in the dispensary so I have to - I’m the only one doing the dispensing. […] if you have to put an order away and put your scripts away and all of those stuff, you don’t have time to be outside [providing extra services]. And I’ve worked in both types of pharmacies...” (Participant 10, locum)*
	Organisational (setting: i.e. community pharmacy sector)	*“Like most people come into the pharmacy and pretty much see it as a supply facility. All this with services is still a little bit with - where did that come from? And particularly when we’re seeing more as a retailer than as a service place, and I think that’s one of the problem in general in Australia, is pharmacy is seen as retailers not as health service providers.” (Participant 1, owner/consultant)* *“I think professionally, when you’re - particularly in the more remote community, I think, the more you’re more highly regarded within a community as someone, you know, the town often will see: you’re an important part of that community and you’re someone that they’ll go to if they’re not well, or they’re sick, particularly in towns that might only have flying or driving health service, you know, doctors and clinics. And they might only, they might have nurse-led health services, so pharmacies can be seen as, you know, an easy first access point if someone’s not well.” (Participant 4, owner/academic)*
**Implementation**	Organisational - c*onsistency*	*“Yeah, most of the time their response is, “Oh, yeah, I’m so sorry. I, you know, I did my interventions but I didn’t do my Medschecks. And oh, I’ll do better this month.” And you do often notice an improvement though, they’ll, they’ll get their target next time. So, you have that chat, then they’re a lot better for the next few months. […] I don’t know, perhaps it’s because it’s not tied to their, their wage or a bonus, so to speak? You know, they do or they don’t do it – well, it’s the same outcome for them either way.” (Participant 19, manager)*
	Organisational - c*ost of delivery of intervention*	*“So the negative is probably – like I find, particularly for HMRs and the RMMRs too, is the remuneration, I find, is not really covering the cost of doing it.” (Participant 1, owner/consultant)* *“I mean if we can do something for a patient that is going to be of benefit to them - of significant benefit to them - then we will go above and beyond to achieve that for them. The thing is that sometimes, it’s not a profitable thing for us, and we will accept that to a certain degree, because we just rely on the pharmacy to absorb the cost and we go, “Well we’ll write it up.””(Participant 6, owner)*
	Organisational -*Modifications*	*“Look, I think that probably the pharmacist - the staffing of pharmacists, you’d have to say, would impact it the most. If you’re starting at one, and you know, you can’t do it - we need at least two. And because we’re a busy pharmacy. […] So I guess that may be why but I wouldn’t underestimate the importance of having the layout and the areas to formally services. I think that is, even when we had the right number of staff to do it, we still failed. And it wasn’t until we completely changed the layout of the store, you know, put the automation in that, that we began to gain some traction with health services.” (Participant 3, owner)* *“And also I don’t want people to feel like they’re being interrogated. It’s not a formal - it’s not like a job interview. Yeah. So I want to remind them just like, ‘Hey it’s just me...’” (Participant 6, owner)*
**Maintenance of intervention effects**	Individual	*“You know, if we were providing them with a staged supply, well then, they’re not seeking any other drugs from anywhere else, you know. Like, for benzodiazepines, for example. That is a good outcome for them, for that particular person. You know, it’s not mean - it doesn’t mean that all they have to have less of it every week or something like that. It just means, at the moment they’re stable. Once they’re stable, then they can start planning things: maybe cutting down on the amount of tablets, and other things. You know, each person has different outcomes that are important to them.” (Participant 8, former locum)*
	Setting	*“And I suppose, you know, another impact of that, of that sort of chronic lack of resource that just exists in most remote places, is that - we, one of our roles is to be there for our patients in our community, and, and that’s a really really important aspect of what we do.” (Participant 7, owner)*

#### Reach to target population

Services were reported to be accessible due to embeddedness in community and prior good relationships with pharmacy staff.

From their reports, the reach of CPS appeared to be mediated by customer expectations of pharmacist roles, access to other health services (e.g. GPs), remoteness, pharmacist workload (including the typical case burden per patient for the area), and the convenience of both pharmacist and client.

The following patient attributes were mentioned often in relation to target populations:

- Rural/remote customers with poor accessibility to other health services
- Lower socio-economic circumstances
- Aboriginal/Indigenous populations
- Low health literacy or reading skills

#### Efficacy or effectiveness in target population

Factors in customer recruitment included differing expectations of pharmacist roles, patient eligibility, ability to pay for private services, receptivity to pharmacist intervention, legal scope, protocol questions that were not comprehensible to consumers, and the perceived formality of consultations in the pharmacy or doctor’s clinic.

Services mentioned in regards to effectiveness included:

- Triage, especially when other health services were unavailable or overloaded
- Case conferencing with the patient’s doctor regarding queries, medical or practical treatment issues on behalf of the patient
- Correcting inaccurate information gathered by patient on the internet
- Gathering accurate medical histories
- Giving patient realistic expectations of treatment, management and treatment goals through the explanation and provision of health information on diseases and new medications
- Affirming correct management of disease by patients
- Solving medical logistical problems for ‘grey nomads’ and rural customers in remote areas through local connections
- Identification of underlying, previously undiagnosed medical issues
- Corrected use of inhaler devices
- Referral to health professionals who are known to provide high quality service
- Personalised explanations of the Australian healthcare system, giving patient the options for accessing relevant services
- Immunisation services
- Medschecks, Pain Medschecks and HMRs
- Hb1Ac testing for time-poor patients
- myDNA nutritional and medication genetic testing
- Monitoring blood pressure

Outcomes reported included:

- Timely access to hospital services, preventing mortalities
- Increased engagement with health services
- Increased health literacy
- Personalised action plans involving doctor collaboration
- Improved communication with collaborating health professionals
- Increased patient rapport and trust
- Improving patient quality of life
- Improved physiological values (e.g. blood glucose, weight, HbA1c, cholesterol)
- Maintenance of patient kidney function
- Decrease in drug-seeking behaviours leading to stabilisation of mental health
- Improved or continued adherence to medication treatments
- Herd immunity
- Decreased hospitalisations and mortality
- Expressions of appreciation and gratefulness for care provided (e.g. flowers, cookies, letters, presents given in the street)
- Improved pharmacist knowledge and motivation to provide services, which also improved motivation to continue in their pharmacist careers

#### Adoption (Organisational level of impact)

##### Staff

Many adopted services were described, which presented different challenges for the staff tasked with administering them.

First, there was challenge in financing the recruitment of additional pharmacists to provide these services, since many new CPS could not be multi-tasked (e.g. Medschecks, HMRs, health screening) due to the need for patient privacy. Many CPS required pharmacist administration.

Second, proprietors and consultant pharmacists alike mentioned a shortage of pharmacists, particularly in rural areas, even if they had the funds to employ additional staff. Government remuneration was deemed inadequate to cover the costs of these extra services, particularly in rural and remote areas which had higher case burdens and longer distances to travel. Service provision was therefore either not possible, or of low quality because inadequate remuneration had resulted in under-resourcing of new CPS. This made pharmacists required to reach target key performance indicators (KPIs) frustrated and angry. Managers, staff and locum pharmacists therefore spoke of excessive workload limitations and frustration with management, particularly if they were attempting to create bottom-up change. They tended to demonstrate this by voicing a sense of powerlessness, dissatisfaction and intention to change jobs or career. Often, they distanced themselves from the pharmacy, in contrast with proprietors. Pharmacist proprietors, instead, tended to relate to their pharmacies as an extension of themselves and their professional values, perhaps due to their financial interest and leadership position. In general, they demonstrated greater power to change their circumstances, trial strategies for CPS success, and employ necessary personnel. On the other hand, pharmacists who were actively engaged and highly motivated in providing CPS spoke of wanting to help patients, increasing personal clinical knowledge, reports of good CPS health outcomes for patients, and of finding alternative funding sources for CPS provision.

##### Community pharmacies

Pharmacies which prioritised and believed in investment in pharmacist wages tended to report a high level of innovation, CPS routinisation and sustainability. Those who perceived CPS adoption was essential to the survival of pharmacies also tended to be more innovative, believing they were preparing for the future and providing for their communities. This manifested as investment into areas such as: innovations to discover what would make CPS sustainable for their businesses, specialised training of pharmacists and staff for CPS, and hiring of more pharmacists rather than pharmacy assistants (if personnel could be found).

Rural and remote pharmacies spoke of their high caseloads per patient, and general resource limitations which meant many could not meet guidelines for more advanced CPS such as Medschecks. However, some spoke of planning and collaborating with other health services to attract and keep a pharmacist workforce for their communities.

Pharmacies espousing high community involvement also reported a high patient caseload due to increased and intensive interactional work, and more involved collaboration with other health professionals. They also generally desired to adopt more CPS regardless of their current services, but reported a lack of financial return for doing so in the current circumstances. In general, these participants did not speak of additional investment into staff, training or exploration of innovations, perhaps because they appeared to be experiencing work overload already.

##### Community pharmacy sector

Adoption in the community pharmacy sector seems to be varied and the sustainability of providing CPS could be perceived to be controversial, due to:

- Participant reports that ‘society doesn’t understand what pharmacists do’, implying a possible limit to patient willingness and ability to pay for CPS. From these accounts, in general, the sector seemed to be reluctant to invest in CPS implementation and innovation;
- Perceived pressures within the sector caused by different business models (e.g. retail models vs patient care models) which may affect societal expectations of pharmacists;
- Legal restrictions in the profession which limit certain tasks to pharmacists, rather than other pharmacy staff;
- Workforce health issues that are implied to cause personnel shortages, and the limitations of the profession to respond to these; and
- Difficulties in role transition from pharmacists as medication suppliers towards being primary health care service providers.

However, embeddedness and personal attachment to surrounding communities appeared to motivate pharmacists towards better clinical care.

#### Implementation

##### Consistency

Not much information was given about the consistency of CPS provision. Disagreement between employees and employers on the importance of CPS provision was mentioned to affect consistency with which they were delivered. One participant suggested employee pharmacists were less concerned about providing CPS consistently since it was not tied to their individual wages.

However, there may be a positive feedback loop for those pharmacists who reported regularly providing CPS: apparently it increased their clinical knowledge and interpersonal skills, which in turn motivated them to continue providing CPS consistently.

##### Cost of delivery of intervention

*Organisational cost.*

Organisational costs included logistical transport and problem-solving, extra staff time necessary for educating illiterate or low health literacy patients who often had no money to pay for services, extra staff and pharmacists, possible dissatisfaction and displeasure of clientele through introduction of services, unexpected administrative burdens, investment in services that patients may not desire, buying equipment (e.g. screening machines, automated dispensing robots), staff training, store layout changes and changes in workflow and business model adjustments.

Some pharmacists asserted that given all these changes, remuneration for service provision was insufficient to cover organisational costs.

*Financial cost.*

Insufficient organisational remuneration for CPS was said to negatively affect employers’ capacity to implement or hire pharmacists to provide a consistent service offering. Participants also reported CPS provision did not reward employees for their extra efforts, whether through sufficient organisational remuneration or individual wages.

*Human cost.*

Despite enjoying increased satisfaction, competence and patient appreciation for ameliorated health outcomes, participants reported extra psychological strain when providing services. On top of these challenges, when faced with unsupportive management and perceived unethical norm for CPS provision, one pharmacist mentioned leaving a permanent position in favour of a ‘flexible’ locum job with more professional autonomy, less responsibilities and decreased pressure. One pharmacist spoke of their proprietor worrying about client receptivity to services, and a possible lack of return for investment into CPS innovations.

##### Modifications

*Store management modifications.*

Pharmacists spoke of changes in store management, layout, staffing and equipment in order to provide more services, such as: joining pharmacy chains that provided support for service implementation or access to private health funding, achieving the correct staffing mix (including pharmacists), changing store layout to include consulting rooms and counselling spaces, installing dispensary automation, setting service targets per time period, ‘sharing’ a dedicated services pharmacist between different stores, offering a compounding service to fill a local need, and trialling clinical knowledge and personality tests during pharmacist job interviews.

*Modifications to approaching patients and providing services.*

Some pharmacists spoke of efforts to make patients comfortable by using informal lay language where possible, and taking the time to translate protocol and medical wording into normal vernacular. It was suggested more formal approaches to CPS were more appropriate for patients the pharmacists were unfamiliar with, whereas more informal consultations with ‘a cup of tea’ (P6) could be carried out with known clientele.

Other modifications included: requesting patients to inform their doctor prior to CPS provision, and instating fees for screening services (e.g. cholesterol panels, blood pressure checks, blood glucose testing).

#### Maintenance of intervention effects

##### Individual

*Maintenance of individual clinical outcomes reported included:*

Maintenance (rather than worsening) of kidney function, decreased drug-seeking behaviour, continued adherence to medication treatment, improved medication management, immunity through vaccination, and giving patients control over their health issues (opioid overuse, mental health issues, chronic pain) through information provision and counselling.

*Maintenance of individual interactional outcomes included:*

Increased patient trust, respect, loyalty and relationships with pharmacists.

##### Community Pharmacy setting

Pharmacies who seemed to maintain CPS (and their effects) were spoken of and represented by those pharmacists reporting a high commitment to service provision, including their trials of different strategies to maintain CPS. CPS, in these cases, aligned with the pharmacy organisational values such as: supporting the whole community to get better health outcomes, provision of information about the quality use of medicine, and bridging socio-economic health disparities in remote/rural and low socioeconomic areas.

In general, CPS were not implemented in the community setting to the extent the pharmacists would prefer.

## Discussion

This exploration of the translation and implementation of CPS from Australian pharmacists’ perspectives demonstrates the complexity of the pharmacist’s role in the community sector. Although these findings report pharmacists views alone, their perceptions as service providers represent a breadth of ‘insider’ experiences which underpin their individual service provision directly. These insights are a strong reminder that pharmacist service providers are also people: their integral part in the healthcare system already requires a high level of accuracy and detail in their work [86, 87], putting them at risk of developing maladaptive perfectionism and related mental illnesses [88, 89]. .To avoid further work strain associated with CPS provision [1, 87, 90–92], an effective implementation of new health services should include evidence-based support and resourcing of providers on every level.

### CPS provision from the pharmacist provider’s perspective (micro level)

#### Services

Although pharmacists made an effort to stay on the topic of service provision, it soon became clear that they often could not separate discussion of enhanced and extended service delivery from more traditional or ‘core’ services such as dispensing.

Nevertheless, the viability of CPS seemed to be a key consideration for pharmacists, likely due to the implications on their jobs and consequences for patients. A reported reliance on governmental remuneration for some rural pharmacists was consistent with the Pharmacy Financial Survey commissioned by the Department of Health, which stated that pharmacies located in Pharmacy Access/Remoteness Index of Australia (PhARIA) zones 3–6 were more likely to rely on funding [93]. (PhARIA describes pharmacy service accessibility and geographic remoteness for areas in Australia; categories of 3–6 include very remote, remote, moderately accessible areas.) [93] This was said to be due to low health literacy in these regions, coupled with the regional tyranny of distance and workforce shortages. These areas also typically had the greatest socio-economic disadvantage: patients probably could not afford payments for extended or enhanced services, or private health insurance. Since both patient service fees and private health insurance repayments were alternate funding streams utilised by a minority of pharmacies to invest in the recruitment of pharmacists, pharmacists in remote and disadvantaged areas reported they could not provide CPS conventionally either, which made them ineligible for government funding.

However, they said stronger patient relationships and poor access to other health services meant that whatever CPS they *could* deliver had greater impact. Although rural pharmacy funding may enable such pharmacies to exist in a one-pharmacist capacity, if public health initiatives are expected to be widely provided in underserved communities, flexible service funding models may be necessary to more completely service remote/regional areas or populations with high socioeconomic disadvantage. This is consistent with a study in the UK which examined pharmacist work in hyperdiverse London communities, which demonstrated a higher caseload burden per patient [55].

#### Patient characteristics

Pharmacists engaged in regular CPS spoke of an increased CPS effectiveness associated with ‘knowing’ the patient beforehand, suggesting a similar effect to that reported in nursing. This dyad between the patient and pharmacist, like that between the nurse and patient, allows the person to be treated and understood as an ‘unique individual’ with selected care choices tailored to the patient. This involves comprehension of the patient’s emotional and physical condition, and their experiences, behaviour and perceptions towards previous health interventions [94]. This is consistent with previous research examining ‘knowing the patient’ with American clinical pharmacists working in anticoagulation outpatient clinics. In a similar fashion, the pharmacists in that study demonstrated ‘knowing the patient’ through their identification of unmet patient needs, explanation of medication treatment other than anticoagulation therapy, and assisting the patient to navigate the healthcare system [95]. Participant 24 spoke of a desire to spend longer times with patients for these reasons. It could be, therefore, that pharmacist involvement in clinical matters through CPS provision facilitates such outcomes which perform a preventative health function.

#### The pharmacists themselves

It appeared that foremost in the minds of these pharmacists was improving the health of their patients, which was ensured by performing services at a high standard of quality. This was linked with feelings of achievement, engagement and motivation. In turn, perceptions of quality of care were affected by workload, pharmacy environment and employer pressure; compromise or quality decline was associated with dissatisfaction with their job and the profession. This may point to the importance of professional identity and perceived organisational support in CPS delivery [96], as service *quality* appeared to change the perceptual meaningfulness of pharmacist work. It is possible that services delivered with inadequate quality could cause individuals to distance themselves from their workplaces due to an apparent incongruence in values, thus causing a decline in organisational commitment and possible intention to leave [96–98]. It could be that pharmacists see themselves as individual and independent clinicians constrained by the pharmacy setting they work in, rather than conceptualising themselves within a strict employer-employee relationship.

Interestingly, pharmacists associated extended and enhanced service provision with possible future career advancement or promotion. Although perceptions of increased job satisfaction from better patient interactions and health outcomes were to be expected from past research [99–104], there was a seemingly unanimous positive outlook on increased recognition and career pathways of pharmacists as health professionals, regardless of their role descriptions. This positivity in the future of the profession is consistent with the UTS Pharmacy Barometer 2018 and 2019 reports [105, 106], and could be due to recent alignment of prominent professional bodies in support of advanced and extended pharmacist roles [31, 32]. The frequency of verbal abuse, robberies, physical aggression, sexual harassment/assault and threats in Australian community pharmacies [107] could be a major driver for the reported pharmacist desire for greater respect and recognition from the wider public and health professionals.

#### The pharmacist workplace

Some participants complained of difficulty in recruiting pharmacists required for service delivery, particularly in regional areas. This was inconsistent with industry talk of pharmacist oversupply [108] and a record number of registered pharmacists in Australia [83]. However, their anecdotal reports are confirmed in governmental statistics from the Department of Employment, Skills, Small and Family Business [109]: retail pharmacist shortages are apparent in metropolitan and regional New South Wales, Northern Territory, South Australia and Tasmania, whilst regional shortages are present in Victoria, Western Australia and Queensland. As reported by the participants, it could be that a significant and unseen quantity of experienced pharmacists are opting out of the profession, possibly due to difficult working conditions, long hours, stress and dissatisfaction [87, 92, 98, 110–115]. This is consistent with a previous study which reported registered pharmacists in Australia leaving the profession due to dissatisfaction, as well as a perceived lack of opportunity for career progression, under-use of pharmacist knowledge and skills and insufficient patient involvement [116]. In the long-term, CPS implementation may require employers to change business models, e.g. restructuring their workforce and pharmacist roles to incentivise pharmacists from an organisational level [117], rather than expecting professional satisfaction and organisational pressure to compensate for the increased strain associated with CPS.

### Implementation on the meso and macro levels

#### Meso level

A lack of agreement on what ‘services’ are and pharmacist difficulty in remembering what CPS were available could have implications for service consistency. Areas of high socio-economic disadvantage and remoteness could also complicate CPS consistency due to related high workloads per patient, which may impact practitioner health.

In general, it appears the approach of expecting pharmacists to implement CPS into their pharmacies has not been effective, since they lack implementation expertise. Training available from professional associations may also be inadequate, particularly if top-down implementation is assumed to be possible, but is not; power structures in pharmacy organisations can complicate matters [98].

Instead, pharmacist proprietors and employees implementing CPS could benefit from ongoing support from an external body with implementation and translation expertise that is based in current academic and organisational research. This should be in preference to assistance from various bodies who may not specialise in the area, or who utilise corporate data. This is important in Australia, where pharmacies are mostly small businesses owned by pharmacists: based on previous research, it may be inappropriate to apply organisational strategies founded upon large corporation data to community pharmacists [1, 15, 118, 119].

Providing targeted implementation support may free pharmacists to better leverage the clinical risks and benefits associated with CPS, and perhaps enable pharmacies to improve their service uptake, consistency and quality, pharmacist working conditions and patient health outcomes.

#### Macro level

Participants pointed out the fundamentally different nature of product supply and CPS delivery, and argued these activities should not be treated the same (i.e. could not be measured by quantity alone). There are measures in place to assure quality of pharmacy services: the Quality Care Pharmacy Program (QCPP) audits Australian pharmacy administrative processes, which includes a cursory check of CPS administration [13, 35], and the Pharmacy Programs Administrator has introduced an auditing process for fraudulent or incorrect claims to allow pharmacists to report rorting, requiring pharmacies to keep all clinical data for 7 years for these audits [120]. However, there are no known *clinical* CPS audits being performed. Instead, the majority of the claims data sent directly to the government or program administrators involves administrative (rather than clinical) data [37]. This lack of clinical (rather than administrative) auditing could lead to doubts about CPS benefits, as other healthcare groups and the government have questioned in the past [13]. Similarly, pharmacists spoke of their doubts about the acceptable standard of CPS, and seemed to desire that pharmacists in other pharmacies would be held to higher accountability. As some suggested, the need for ongoing accreditation in specific CPS and clinical audits may be welcomed by the community and profession to guarantee an acceptable standard of care. This data was consistent with previous government audits into CPAs, where pharmacists valued specific training and accreditation [16, 18, 33, 36].

It would be amiss to address the implementation of CPS without acknowledging the ongoing role transition of pharmacists from a product supply role towards a health service provider role [56]. This is important because CPS implementation represents a change in work expectations, tasks and competencies in pharmacies. However, the professional identity of a pharmacist may now favour health service provider roles, whilst their organisational pharmacist roles still favour product supply tasks. These conflicting expectations can only be balanced by pharmacy organisations aligning with one of these identities and providing congruent rewards [96, 98, 121–123]. When the professional identity, rather than traditional supply roles, is rewarded for fulfilling health service provider tasks and expectations, it may allow pharmacists to implement patient-centred care and innovate in CPS implementation [1, 41]. Conversely, rewarding product supply roles incentivises individual pharmacists to remain in more traditional forms of pharmacist work, and avoid CPS provision and implementation.

Since Australian pharmacists are expected to practise ethically and independently of employer expectations, pharmacy owners should avoid punishing or pressuring their employees to provide CPS. Instead, the structural support required for a customised approach towards pharmacist role transition could be adopted in pharmacies desiring to implement greater CPS offerings. This would involve rewarding quality CPS provision and implementation, and incentivising training in clinical and implementation skills (e.g. bonuses for providing quality CPS at measurable, achievable targets; funded training for CPS delivery and pharmacist specialisation; etc.). Measures of pharmacist productivity, too, require revision if CPS implementation is to be taken seriously. For example, prescription numbers are hardly an appropriate measure for the productivity of a pharmacist if they are not the dispensing pharmacist; in a similar way, quantities of Medschecks provided are barely adequate productivity measures if their outcomes are poor (i.e. not applicable or acceptable to other health professionals) and not well-founded clinically (which may cause conflict between doctors, pharmacists and patients) [16, 18, 33, 36].

### Limitations

Of course, this qualitative study is not representative of all community pharmacists in Australia due to its sample size and study design. However, the sample of participants was roughly comparable to AHPRA statistics on pharmacist representation.

It must be acknowledged that due to initial purposive sampling, participants were likely to be engaged, positive and motivated in their pharmacist work, which lent a positive note to most of their words. However, several disgruntled and frustrated pharmacists were included in the sample due to recruitment from other avenues, and thus gave different perspective to their experiences of CPS implementation and provision, and insight into pharmacy power structures. In order to give voice to these perspectives, another manuscript relating positive, negative and neutral pharmacist stances towards CPS and work strain is soon to be published.

The researcher (FY) who conducted, transcribed and analysed the interviews is a current doctoral candidate at UTS and a registered community pharmacist. However, this was an advantageous factor in the recruitment process since Australian pharmacists were difficult to recruit and engage with research [124]. Further engagement with industry bodies is planned.

Rather than specific strategies of establishing rigor in this study [125], the following were used to interrogate researcher/participant bias and establish reflexivity [71]: field notes, researcher journaling, regular debrief meetings with academic supervisors and informal discussions with pharmacist peers about study findings. These were used over a period of 15 months after the study was held. Additionally, the findings into the pharmacist micro perspective were presented by FY at a Hobart pharmacist conference in February 2020. Informal discussions with pharmacist audience members after the presentation confirmed findings.

These measures led to further recognition of the disparate manner in which CPS are implemented in Australia, the role of professional associations in the implementation process, and the acknowledgment that pharmacist proprietors may also feel powerless in their pursuit of business viability and patient care. There was acknowledgement that Australian pharmacy academics, despite their best intentions, are likely to have suffered disappointment in the translation of CPS into practice. Thus turning from fault-finding, this paper sought solutions towards better CPS provision instead.

Finally, although operationalising the RE-AIM framework for each individual CPS mentioned in the data would be ideal, this was beyond the scope of this paper. For example, during interviews, pharmacists often were unable to remember all the services their pharmacy provided, due to the sheer quantity involved. Within the named services alone (and not including general dispensing services), there were 16 services which were reported by participants in total. Each of these are associated with respective guidelines, protocols and potential target populations, and would require further investigation for understanding actual implementation in community pharmacies.

### Future directions

Evaluation using implementation science frameworks such as RE-AIM for individual CPS of interest could be insightful, since some skew towards product supply and administration (e.g. compounding, staged supply, vaccination), whilst others lean towards health coaching (e.g. weight loss and smoking cessation programs), and still others towards health screening and promotion (health checks, HbA1c screening, triage). Wound care services, for example, can also span the three former categories since some pharmacies include bandage supply in their service fee. Whereas some CPS have program guidelines articulated in a protocol, others (e.g. clinical interventions, requests for pharmacist-initiated therapy) do not have formalised protocols and instead rely on the professional knowledge of the pharmacist to use *clinical* protocols and guidelines instead, which can be at odds with organisational goals and cause frustration. Thoughtful categorisation and evaluation of the plethora of different CPS being offered in Australian pharmacies could shed more light on what types of services are easily implemented in the current pharmacy landscape, the types of services which require simple organisational interventions in order for successful implementation, and the services which necessitate third party support and government funding.

Lastly, it could be that professional practice models, such as those used in nursing practice models, could be helpful organisational tools to address both role transition and implementation. These models not only contain professional and role expectations, but also explain the interactional and financial engagement required for the sustainability of new initiatives [126]. Rather than simply expecting pharmacists to implement CPS into a community pharmacy model of working, this could be a new area of research where case reports of successful and sustainable practice models could be expanded upon, tested and verified in different communities. These professional practice models recognise the different subsystems operational during clinician work, and ensure higher levels of direct applicability to workplaces [126].

## Conclusions

Community pharmacists reported CPS translation difficulties in the Australian setting. They disagreed on the definition of CPS, whether new services were fundamentally different to those already being provided, and found it difficult to remember all the services they provided. Although some demonstrated ongoing efforts to provide CPS with varying success, these front-line clinicians were under-resourced and trained to action implementation and change management processes in their setting, since their primary occupation and workload was in patient care delivery. Given that Australian community pharmacies are often small businesses, it may be necessary to investigate different implementation strategies that support ongoing pharmacist role transition, provide training and resources for individual clinicians and pharmacy organisations, and ultimately reward the clinical and interactional quality of services delivered.

## Data Availability

The datasets generated and/or analysed during the current study are not publicly available due to its sensitive commercial and private nature, but are available from the corresponding author on reasonable request.

## APPENDIX 1

### Participant demographics

**Table:**

			Pharmacy type	#, (% of community jobs held)*
**Male (%)**	12 (52.2%)			
**Female (%)**	11 (47.8%)		Independent	13 (46.4%)
Education			Franchise/banner	14 (50.0%)
**Masters (%)**	8 (34.8%)		Friendly society	1 (3.6%)
**Bachelor (%)**	15 (65.2%)		*Location*	
*Years since registration*	**# (% of total participants)**		**ACT**	2 (8.7%)
**0–9**	11 (47.8%)		**NSW**	10 (43.5%)
**10–19**	6 (26.1%)		**QLD**	2 (8.7%)
**20–29**	3 (13.0%)		**TAS**	1 (4.3%)
**30–39**	2 (8.7%)		**VIC**	6 (26.1%)
**40+**	1 (4.3%)		**WA**	2 (8.7%)
*Years in main pharmacist job*			*MM* [84]	
**1–5**	12 (52.2%)		**1: Metropolitan areas**	12 (52.2%)
**6–10**	7 (30.4%)		**2: Regional centres**	–
**11–33**	4 (17.4%)		**3: Large rural towns**	1 (4.3%)
*Age*			**4: Medium rural towns**	1 (4.3%)
**24–29**	5 (21.7%)		**5: Small rural towns**	5 (21.7%)
**30–39**	11 (47.8%)		**6: Remote communities**	1 (4.3%)
**40–49**	4 (17.4%)		**7: Very remote communities**	1 (4.3%)
**50–59**	1 (3.6%)		**Not given**	2
**60–69**	2 (8.7%)			
			IRSD/SEIFA [85]	Participants
*Pharmacist jobs represented**			5: Least disadvantaged	3 (13.0%)
**Owner**	9		4	3 (13.0%)
**Consultant**	3		3	4 (17.4%)
**Staff**	6		2	3 (13.0%)
**Locum**	3		1: Most disadvantaged	5 (21.7%)
**Manager**	**3**		No ABS data	3 (13.0%)
**Casual staff**	2		Missing	2 (8.7%)
**Hospital staff**	4			
**Academic**	1			
**Professional association staff**	1			

*Total is greater than 23 as around one third of participants had multiple pharmacist positions

**Table:**

	LOCATION						
State	Participants	PhAria [127]	Participants	MM [84]	Participants	IRSD/SEIFA [85]	Participants
**ACT**	2 (8.7%)	**1: Highly accessible**	13 (56.5%)	**1: Metropolitan areas**	12 (52.2%)	5: Least disadvantaged	3 (13.0%)
**NSW**	10 (43.5%)	**2: Accessible**	–	**2: Regional centres**	–	4	3 (13.0%)
**QLD**	2 (8.7%)	**3: Accessible**	4 (17.4%)	**3: Large rural towns**	1 (4.3%)	3	4 (17.4%)
**TAS**	1 (4.3%)	**4: Moderately accessible**	2 (8.7%)	**4: Medium rural towns**	1 (4.3%)	2	3 (13.0%)
**VIC**	6 (26.1%)	**5: Remote**	1 (4.3%)	**5: Small rural towns**	5 (21.7%)	1: Most disadvantaged	5 (21.7%)
**WA**	2 (8.7%)	**6: Very remote**	1 (4.3%)	**6: Remote communities**	1 (4.3%)	No ABS data	3 (13.0%)
		**Not given**	2 (8.7%)	**7: Very remote communities**	1 (4.3%)	Missing	2 (8.7%)
				**Not given**	2		

## APPENDIX 2

### Data collection form

ETH19–3471: FACTORS WHICH AFFECT AUSTRALIAN COMMUNITY PHARMACIST SERVICE PROVISION.

PARTICIPANT SURVEY

Date of interview:_________________.

1. Personal details

Age: ______________.

What gender do you identify as? Male Female Other.

2. Pharmacy career

Year first registered as a pharmacist: ______________ Years in current (main) job: ________________.

Highest degree of education: ________________________.

3. SELF-EMPLOYMENT/EMPLOYEE? *Please tick all that apply.*

Are you:

□ employee at a community pharmacy

□ pharmacist employee at other setting

□ self-employed pharmacy owner(please specify): _____________________

□ self-employed locum□ self-employed at other setting

□ self-employed consultant pharmacist (please specify):_____________________

□ other (please specify):________________

4. Sector of practice

Please fill in the table below using given descriptions for job description & sector; if none are applicable, please use the “other” options and specify.

**Table:**

	Job description 1. Owner 2. Pharmacist Manager (manages a team including pharmacists) 3. Locum 4. Staff 5. Consultant 6. Other (*please specify*)	Sector 7. Community, 8. Hospital, 9. Consultancy, business 10. Primary care, organisation 11. Industry, 12. Academia, 13. Medical clinic, 14. Other pharmacy (please specify) 15. Other non-pharmacy (please specify)	Average weekly hours
**Job 1**			
**Job 2**			
**Job 3**			
**Job 4**			

5. If you ticked one (or more) of the above categories within community pharmacy, please indicate the pharmacy organisation type

Job 1

□ Independent pharmacy

□ Franchise/banner pharmacy

□ Friendly society pharmacy

Job 2

□ Independent pharmacy

□ Franchise/banner pharmacy

□ Friendly society pharmacy

Job 3

□ Independent pharmacy

□ Franchise/banner pharmacy

□ Friendly society pharmacy

Job 4

□ Independent pharmacy

□ Franchise/banner pharmacy

□ Friendly society pharmacy.

6. Please provide the postcodes of the pharmacy/ies that you work in so that we can identify its PhAria category

Job 1 _ _ _ _ Job 2 _ _ _ _ Job 3_ _ _ _ Job 4_ _ _ _

7. For locums only: if you are a locum working in community pharmacy, how many pharmacies do you work in, on average, over a month? ___________________

## APPENDIX 3

### INTERVIEW GUIDE

Thanks for agreeing to be part of this research. We really appreciate your willingness to participate. The reason we are having these interviews is to find out your experiences and opinions about factors that affect community pharmacist service provision. The information provided will help us improve our understanding of what affects a community pharmacist providing services. The results will be used in a nationwide survey that will provide a snapshot of the issues and solutions possible in providing services, and thereby inform pharmacy decision makers in how to best support community pharmacists in their work, particularly in services. We need your input and want you to share your honest and open thoughts with us.

Structure of interview.

The interview will include four primary stages as follows:

**Table:**

Stage of Interview	Role of researcher	Time
Introduction and general participant information	*Researcher will provide an overview of the goals and purpose of the discussion. Participants will answer general questions about themselves for demographic data.*	15 min
Personal opinions on pharmacist jobs, pharmacy, the community sector and profession	*Researcher facilitates a structured discussion on a topic that is easy for participants to answer, to start the talking and sharing.*	20 min
In-Depth Discussion: Internal/external demands, framework, role stresses and strains	*Researcher will ask questions related to the main purpose of the interview, and encourage conversation that reveals the participant’s thoughts and feelings. This is where the key data is collected.*	45 min
Closure	*The researcher will answer any remaining questions from participants, then thank the participants and indicate next steps.*	10 min

Interview rules.

1. We want you to do the talking

This is about your individual experience, and would like you to share as much as you feel comfortable sharing. We do have a lot of questions, but more importantly, we want you to share your experience so that others will benefit from your insights.

2. There are no right or wrong answers

Your experiences and opinions are important and we want to hear what you have to say!

3. What is said in this interview stays HERE

I want you to feel comfortable sharing when sensitive issues come up.

4. We WILL be recording this interview

We want to capture everything you have to say. The session will be recorded but we will anonymise your involvement in the study and details that could be used to identify you.

Interview Guide.

NB: Words in italics are prompts only.

1. What services do you provide personally in the pharmacy?
2. What factors do you take into consideration when providing these services?
  - *For example, internal thoughts? (Prompts: pressure from self, personal resources/knowledge, pharmacist identity, individual characteristics)*
  - *Or individual characteristics? (*e.g. *ethical values, age, people the pharmacist looks up to, and includes what they think a pharmacist should do and shouldn’t do, what preferences pharmacists have in doing specific tasks, and how they are used to working)*
  - *Personal resources? (*E.g. *knowledge, skill, attitude, commitment to role/organisation/career/pharmacy profession, ability to perform the service, including role competence, interpersonal competence and how they present themselves to others*
  - *External factors?* (*Prompts: role partner expectations, legislative limitations, workplace setting)*
  - *Interactions with different people?*
    E.g. *patients, doctors, allied health, pharmacy staff, supervisors, other pharmacists, industry representatives, professional associations, government bodies like AHPRA, pharmacy organisation*
    i. *The most impact?*
    ii. *What impact?*
    iii. *Coping?*
    iv. *The relationship between pharmacy staff and patients?*
    v. *The relationship between the doctor and the patient affecting service provision?*
  - *The pharmacy workplace?*
    i. *Organisational culture = the atmosphere of the pharmacy that evolves over time due to systemic processes, habits and management approaches*
      *Organisational climate = the current atmosphere in the organisation)*
    ii. *Values? (*e.g. *discount pharmacies and service-focused pharmacies; HR practices such as pay, leave and dismissal)*
    iii. Status/authority?
      1. *Part-time, full-time, casual positions*
      2. *The job you occupy (owner, manager, employee, other)*
      3. *Autonomy, or freedom to work how you want to*
    iv. Rewards and punishments?
      1. *Wages*
      2. *Other benefits (*e.g. *salary packaging)*
      3. *Punishments (*e.g. *underperforming or unethical practices)*
    v. Physical resources?
      1. *The physical environment*
      2. *Equipment available*
      3. *Staffing levels*
      4. *Multilingual support*
      5. *Clinical references*
      6. *Stock availability*
      7. *If you had all the resources in the world, what kind of support would you****need****when providing services?*
      8. *What support would you****like****to have when providing services?*
    vi. *Normal working conditions?*
      1. *Workload*
      2. *Type of work activities expected of the pharmacist to do*
      3. *Perception of how good the job is in comparison to alternate jobs*
      4. *Career advancement available (if any)*
      5. *Work hours (including meal breaks)*
      6. *Physical location of the pharmacy (*i.e. *rural, metropolitan)*
      7. *Type of pharmacy it is identified as (*e.g. *discounting pharmacy)*
    vii. *How time is allocated?*
      1. *Tell me about multi-tasking with these activities.*
      2. *Tell me about multi-tasking and service provision.*
      3. *Are there factors which have changed or could change your allocation of time to different activities?*
  - *Factors outside of job? (*e.g. *Roles****at****home (outside their job like being a parent or caring for family members, social factors like time with your friends, and responsibilities in the community or other organisations in the public)*
3. In your opinion, which of these factors you listed impact your service provision the most?
4. What would or does make service provision worthwhile for you?
5. Tell me about your personal view of service provision: do you have any positive, negative, or neutral feelings towards it?
6. Give me your thoughts on whether providing services has personally affected you in any way, positively or negatively.
7. Disregarding your current position in life, if you could do any pharmacist role possible, what would you do?
8. Is there anything you’d like to add or comment on what we talked about today?

## Abbreviations

CPS: Cognitive pharmacy services (58)
CPAs: Community Pharmacy Agreements
3CPA: Third Community Pharmacy Agreement
5CPA: Fifth Community Pharmacy Agreement
7CPA: Seventh Community Pharmacy Agreement
COREQ: Consolidated criteria for reporting qualitative research
DMAS: Diabetes Management and Assessment Service
HMR: Home Medicine Review
MMM: Modified Monash Model
QCPP: Quality Care Program Pharmacy Program
RE-AIM: Reach/Effectiveness/Adoption/Implementation/Modification framework
PSA: The Pharmaceutical Society of Australia
PhAria: The Pharmacy Access/Remoteness Index of Australia
S3: Schedule 3 ‘Pharmacist Only’ Medicines of the Australian Poisons Standard, the Standard for the Uniform Scheduling of Medicines and Poisons (SUSMP)
‘The Guild’: The Pharmacy Guild of Australia
UTS: University of Technology Sydney
